# Diagnostic performance of a RHAM-based point-of-care test for *Mycobacterium tuberculosis*

**DOI:** 10.3389/fpubh.2025.1663233

**Published:** 2025-11-20

**Authors:** Xiang Chen, Giovanni Ladu, Vincenzo Lai, Leonardo Antonio Sechi, Paola Molicotti

**Affiliations:** 1 Health Care Center, The First Affiliated Hospital of Shantou University Medical College, Shantou, Guangdong, China; 2 Department of Biomedical Sciences, University of Sassari, Sassari, Italy; 3 Department of Microbiology and Virology, AOU Sassari, Sassari, Italy

**Keywords:** *Mycobacterium tuberculosis*, laboratory, diagnostic method, point-of-care test, ASSURED criteria

## Abstract

Tuberculosis (TB) remains a global health crisis, hampered by significant diagnostic delays, particularly for extrapulmonary TB and in resource-limited settings. The development of point-of-care tests (POCTs) meeting the WHO’s ASSURED criteria is crucial. This prospective laboratory-based study evaluated the diagnostic performance of a novel, affordable POCT based on RNase Hybridization-Assisted Amplification (RHAM) technology for detecting *Mycobacterium tuberculosis* complex. The test was evaluated using a variety of clinical specimens collected consecutively from suspected TB patients, compared against standard methods (PCR, Microscopy, culture). The RHAM-based POCT demonstrated promising sensitivity of 83.3% (10/12; 95% CI: 50.9–97.1%) and a specificity of 100% (25/25; 95% CI: 83.4–100%). All five non-tuberculous mycobacteria samples were correctly identified as negative. The two false-negative results occurred in samples with very high PCR cycle threshold values (>36), suggesting detection challenges in paucibacillary specimens. The test exhibited a rapid average turnaround time of 18 min and requires minimal infrastructure, operating via a portable, low-power consumption device, even compatible with mobile phone or car chargers. Its closed-cartridge system enhances biosafety by minimizing aerosol generation. Furthermore, the estimated cost per test is substantially lower than leading commercial molecular assays. This study indicates that the RHAM-based POCT is a rapid, user-friendly, and cost-effective diagnostic tool with high specificity. Its ability to function with diverse specimen types positions it as a potential game-changer for TB diagnosis in field and resource-poor environments, though larger-scale studies are warranted to confirm sensitivity, especially in low-bacterial-load scenarios.

Tuberculosis (TB) remains one of the most pressing global public health threats, with the World Health Organization (WHO) reporting an annual incidence of over 11 million cases worldwide (1). As the leading infectious disease killer, TB claims approximately 1.3 million lives each year, surpassing even HIV/AIDS and malaria in mortality rates (2). A particularly alarming issue is that nearly 30% of TB cases are undiagnosed, which makes them a potential source of infection on the move (1, 2). This diagnostic gap not only leads to treatment delay but also increases the risk of transmission, especially in high-burden regions with limited healthcare access. Accurate and rapid diagnosis is the key to achieving the WHO’s “End TB strategy,” which aims to reduce 95% of TB deaths by 2035. Point-of-care tests (POCTs) enable immediate detection at the community level, offer the chance of early intervention, curb further spread, and simultaneously decrease the risk of TB transmission (3). However, developing an ideal POCT that fully complies with the Affordable, Sensitive, Specific, User-friendly, Rapid and robust, Equipment-free and Deliverable to end-users (ASSURED) criteria defined by WHO is still challenging. Current diagnostic tools often fall short due to technical complexity, infrastructure requirements, or high costs. This study aimed to test the performance of a POCT for tuberculosis diagnosis with a simple procedure, portable device, and reasonable price.

This was a prospective laboratory-based evaluation study. All consecutive clinical samples submitted to the Microbiology and Virology Department of AOU Sassari between August and October 2024 for suspected tuberculosis diagnosis were included in this study. The only exclusion criterion was an insufficient sample volume for performing both the POCT and all reference standard tests. The samples consisted of sputum (*n* = 9), tongue swab (*n* = 1), bronchial aspiration (*n* = 11), bronchoalveolar lavage (*n* = 3), gastric aspiration (*n* = 2), feces (*n* = 2), cerebrospinal fluid (*n* = 1), urine (*n* = 1), and biopsy (*n* = 1). Five extra non-tuberculous mycobacteria (NTM) samples were included for further confirmation of specificity. All the subjects were informed that their samples would be used for Mycobacterium identification. The POCT kit used for TB identification is from Pluslife Biotech Co., LTD. (Guangzhou, China). The test kit is based on the RNase hybridization-assisted amplification (RHAM) technology, a nucleic acid amplification method that utilizes specific probes for the detection of *Mycobacterium tuberculosis* complex DNA. The analyzer is a compact device (dimensions: 101 mm * 91 mm * 65 mm; weight: 210 g). Just a mobile phone charger or a 12 V car charger can power the portable device, making it suitable for use in field settings with unstable or no grid electricity. The entire process, from sample loading to result generation, is fully integrated into a single-use, closed test card. As shown in Figure 1A, sputum and tongue swabs are the manufacturer’s recommended specimens. The sample underwent a 5-min thermostatic vortex mixing before being transferred to a card. The result was generated in 10 to 25 min after inserting the test card into the device, and wireless output to a personal computer or mobile device would be available. Traditional TB identification methods are performed as well, including microscopy smear examination after acid-fast staining, culturing, and polymerase chain reaction (PCR). Lowenstein–Jensen (L–J) solid medium and Mycobacterium growth indicator tube (MGIT) liquid medium were used for culturing. The Anyplex™ MTB/NTMe Real-time Detection Kit from Seegene Inc. (Seoul, Korea) was applied to PCR.

**Figure 1:**
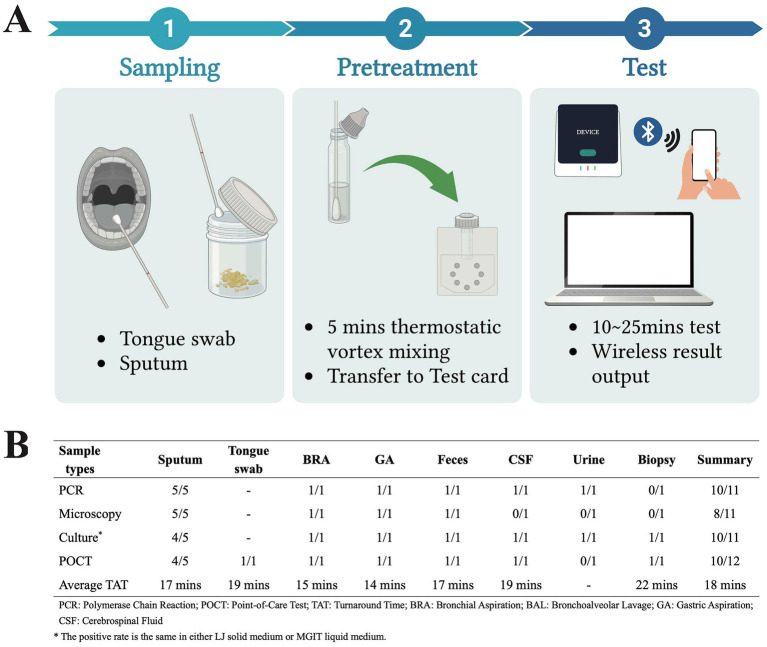
Workflow and results of the RHAM-based POCT (Image created with BioRender).

The POCT showed strong performance in TB diagnosis, across nine sample types beyond manufacturer-recommended specimens (sputum or tongue swab), with only two false negatives derived from sputum and urine (Figure 1B). As for specificity, it is 100% using sputum (4/4), bronchial aspiration (10/10), bronchoalveolar lavage (3/3), gastric aspiration (1/1), feces (1/1), biopsy (1/1), and NTM samples (5/5). The overall sensitivity and specificity of the POCT were 83.3% (10/12, 95% CI, 50.9–97.1%) and 100% (25/25, 95% CI, 83.4–100%), respectively. Statistical analysis using McNemar’s test showed no significant difference in detection rates between the POCT and PCR, or microscopy, or culture(*p* > 0.05). The average turnaround time (TAT) is 18 min from sample pretreatment to results output. About the two false negative samples, they exhibited high PCR Cycle threshold (Ct) values (36.94 and 39.34), while the average Ct value of the other positive samples was 25.65. Notably, the average Ct value of using colonies from the culture medium (18.96) is significantly lower than clinical specimens (27.13) in our daily tests (*p* < 0.01). Thus, we suspected that a low pathogen load in sample underlie detection failure and the pathogen count might be below the limit of detection specified by the manufacturer (50 CFU/mL).

This study demonstrates the promising potential of RHAM-based POCT in TB diagnosis, showing promising sensitivity and high observed specificity across a broad range of clinical specimens, aligning with WHO’s ASSURED criteria for sensitive, specific, and user-friendly. Its rapid TAT (18 min average) and minimal infrastructure requirements position it as a potential solution for resource-limited settings where diagnostic delays perpetuate transmission. Two false-negative results were both associated with very high Ct values, which indicates the correlation between pathogen load and the accuracy of POCT results. This also highlights a common challenge in TB diagnostics, particularly for paucibacillary disease, which is more frequent in children, people living with HIV, and cases of extrapulmonary TB (4). To enhance the sensitivity of the POCT in such scenarios, strategies such as pre-treatment of samples with centrifugation or filtration to concentrate bacilli, or technological refinement of the amplification method to lower the limit of detection, could be explored. While sensitivity requires optimization for paucibacillary samples, the test’s robustness across varied specimens is encouraging. The POCT’s versatility beyond sputum samples expands its practical applicability and also addresses the critical need for extrapulmonary TB detection. Our cost-analysis indicates that the list price of this POCT system (~3 USD per test) is more affordable than that of widely used commercial molecular tests, such as Xpert MTB/RIF (~10 USD per test), potentially enhancing its accessibility in resource-limited settings (5, 6). In terms of biosafety, the RHAM-based POCT offers a notable advantage. Similar to the GeneXpert system, the test utilizes a closed-cartridge system that minimizes the need for manual sample manipulation after initial loading, thereby reducing the risk of generating infectious aerosols compared to open-batch processing such as traditional PCR or smear microscopy. Furthermore, the device’s low power consumption and flexibility in power sources align with the “Equipment-free” and “Deliverable” aspects of the ASSURED criteria, addressing a critical challenge for diagnostic deployment in remote areas. This RHAM-based POCT has demonstrated its advantages in the detection of SARS-CoV-2 and other pathogen (7, 8). While this study also demonstrates the promising performance of the RHAM-based POCT across a variety of sample types, a key limitation is the relatively small sample size, particularly for certain specimen types. To further validate and generalize these findings, future studies involving larger cohorts from tuberculosis-endemic regions are warranted. In summary, our findings support the integration of this POCT into TB diagnostic workflows, especially in field settings where conventional methods are impractical.

## Data Availability

The original contributions presented in the study are included in the article/Supplementary material, further inquiries can be directed to the corresponding authors.
