# Correction: Diagnostic performance of an RHAM-based point-of-care test for *Mycobacterium tuberculosis*

**DOI:** 10.3389/fpubh.2026.1789832

**Published:** 2026-02-23

**Authors:** Xiang Chen, Giovanni Ladu, Vincenzo Lai, Leonardo Antonio Sechi, Paola Molicotti

**Affiliations:** 1 Health Care Center, The First Affiliated Hospital of Shantou University Medical College, Shantou, Guangdong, China; 2 Department of Biomedical Sciences, University of Sassari, Sassari, Italy; 3 Department of Microbiology and Virology, AOU Sassari, Sassari, Italy

**Keywords:** *Mycobacterium tuberculosis*, laboratory, diagnostic method, point-of-care test, ASSURED criteria

Author **Giovanni Ladu** was omitted as an author in the published article. The correct author list reads:

Xiang Chen^1,2^, Giovanni Ladu^2^, Vincenzo Lai^3^, Leonardo Antonio Sechi^2,3^^*^, Paola Molicotti^2,3^^*^

^1^Health Care Center, The First Affiliated Hospital of Shantou University Medical College, Shantou, Guangdong, China

^2^Department of Biomedical Sciences, University of Sassari, Sassari, Italy

^3^Department of Microbiology and Virology, AOU Sassari, Sassari, Italy

The Author Contributions Statement has been corrected to read:

XC: Writing – original draft, Data curation, Visualization. GL: Writing – review & editing, Methodology, Resources. VL: Methodology, Resources, Writing – review & editing. LS: Supervision, Writing – review & editing. PM: Conceptualization, Supervision, Writing – review & editing.

The original version of this article has been updated.

