# Supplementary figures and images for: Diagnostic performance of a RHAM-based point-of-care test for Mycobacterium tuberculosis
Source: Front Public Health. 2025 Nov 20;13:1663233. doi: 10.3389/fpubh.2025.1663233 (PMC12676282; doi:10.3389/fpubh.2025.1663233)

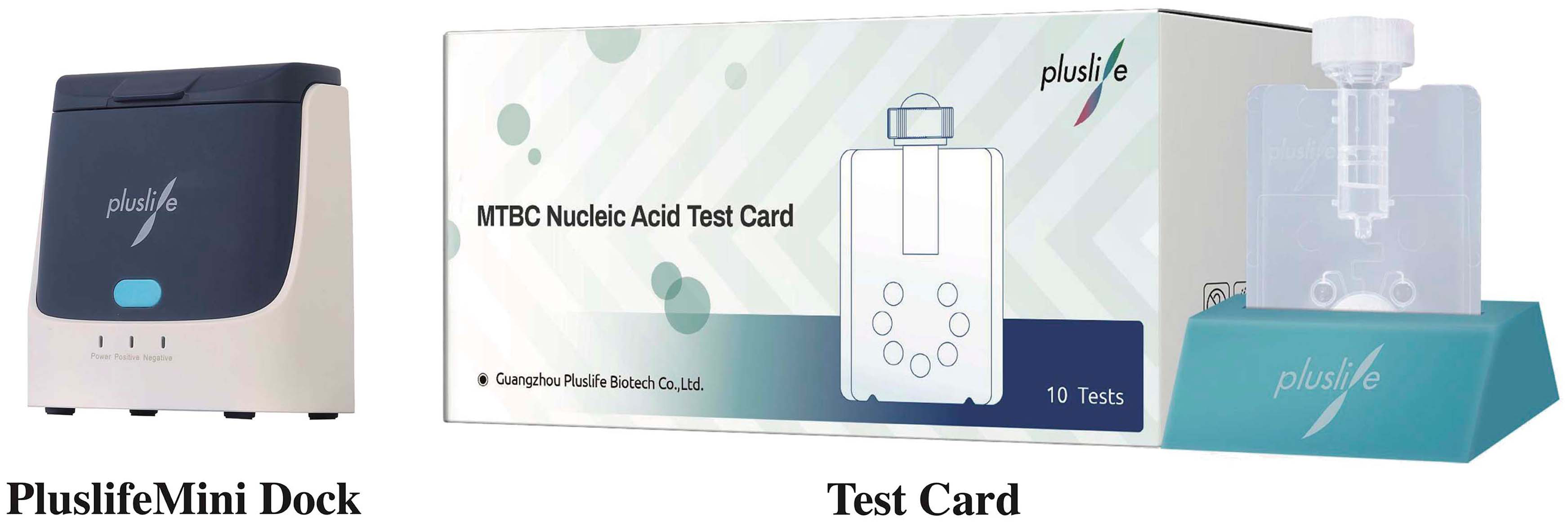

Supplement: Supplementary file 1 [file Image_1.tif]
