# Supplementary material for: Diagnostic performance of a RHAM-based point-of-care test for Mycobacterium tuberculosis
Source: Front Public Health. 2025 Nov 20;13:1663233. doi: 10.3389/fpubh.2025.1663233 (PMC12676282; doi:10.3389/fpubh.2025.1663233)
Supplement: Supplementary file 2 [file Table_1.docx]

| Supplementary table 1. Accuracy of POCT among different sample types | | |
| --- | --- | --- |
| Sample types | Sensitivity | Specificity |
| Sputum | 4/5 | 4/4 |
| Tongue swab | 1/1 | - |
| BRA | 1/1 | 10/10 |
| BAL | - | 3/3 |
| GA | 1/1 | 1/1 |
| Feces | 1/1 | 1/1 |
| CSF | 1/1 | - |
| Urine | 0/1 | - |
| Biopsy | 1/1 | 1/1 |
| NTM | - | 5/5 |
| BRA: Bronchial Aspiration; BAL: Bronchoalveolar Lavage; GA: Gastric Aspiration;  CSF: Cerebrospinal Fluid; NTM: Non-Tuberculous Mycobacteria | | |
